# Traditional Herbal Knowledge among the Inhabitants: A Case Study in Urgam Valley of Chamoli Garhwal, Uttarakhand, India

**DOI:** 10.1155/2019/5656925

**Published:** 2019-06-03

**Authors:** Ankit Singh, Robbie Hart, Sudeep Chandra, M. C. Nautiyal, Alexander K. Sayok

**Affiliations:** ^1^High Altitude Plant Physiology Research Centre (HAPPRC), H.N.B. Garhwal University, Post Box 14, Srinagar Garhwal 246174, Uttarakhand, India; ^2^William L. Brown Center, Missouri Botanical Garden, P.O. Box 299, St. Louis, MO 63166, USA; ^3^Institute of Biodiversity and Environmental Conservation, Universiti Malaysia Sarawak, 94300 Kota Samarahan, Sarawak, Malaysia

## Abstract

The Indian Himalaya is rich in plant species, including many medicinal plants, greatly valued by local inhabitants for health care needs. The study in Urgam Valley of Uttarakhand, India, is to identity and document traditional knowledge of medicinal plants. The study revealed high consensus on medicinal plant usage, with 51 species belonging to 31 families used for local health care. Number of species and uses known increases with age, and elders and specialist healers retain higher levels of traditional medicinal plant knowledge, having unique knowledge of medicinal plants and their uses as well as preparation.

## 1. Introduction

India is rich in floral diversity, with more than 17,000 angiosperm species, 64 gymnosperms, 1,200 pteridophytes, 2,850 bryophytes, and 2,021 lichens [1]. Out of the total, 7,500 species have been reported to have medicinal uses [2]. Diverse topography and climatic conditions provide the Indian Himalaya with an especially rich medicinal plants, whereby alpine areas being the major source of important medicinal plants.

Inhabitants of rural and remote areas still rely on plants as a major component of their health care systems. Indigenous medicines provide considerable economic benefits to local people [3]. The World Health Organization (WHO) mentioned that about 25% of modern medicines are developed from plant sources used traditionally; and research on traditional medicinal herbal plants leads to discovery of 75% of herbal drugs [3, 4]

Locals acquire knowledge of the economic values and medicinal properties of many plants through need, observation, trial and error, and the transmitted experiences of elders. Often, knowledge is concentrated in specialist healers. Most diseases cured by local herbalist are common problems such as respiratory diseases, aches and pains, wounds, and musculoskeletal ailments. Inhabitants often use local medicinal plants without prior advice of local traditional healers because they are using these plants since generations [5]. This knowledge may be passed secretively from one generation to the next through word of mouth [6] or inherited via medico-spiritual manuscripts [7]. Although knowledge of these valuable plants is often restricted within lineages or in other ways, ensuring that the younger generations in these areas acquire this knowledge is essential to its continuity in use and sustainability. Medicinal plant richness of the Indian Himalaya is exemplified in Garhwal Himalaya, within northwestern India. This study focused on traditional herbal medicines of Urgam, a mountain valley in the Garhwal Himalaya rich in medicinal plants which are still used by local inhabitants and specialist healers.

## 2. Materials and Methods

### 2.1. The Study Area

**Figure 1:**
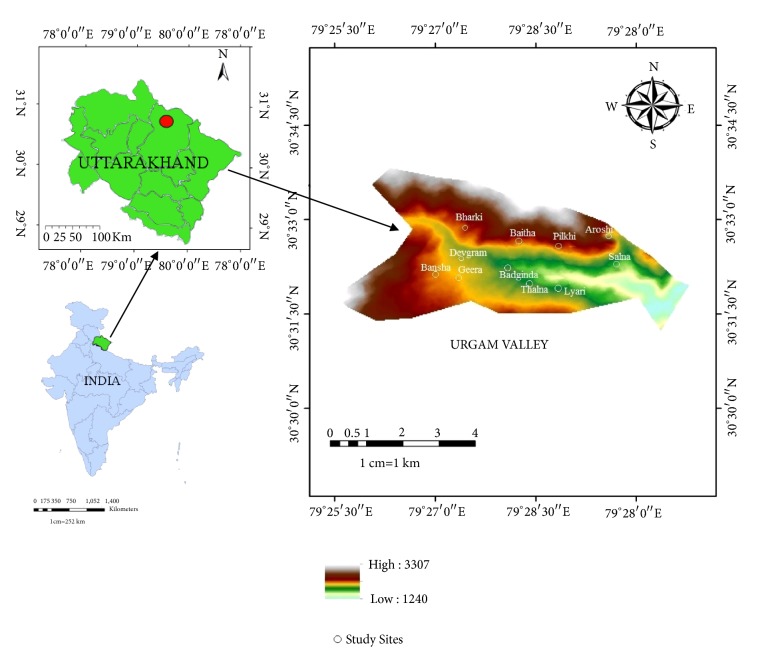
Urgam Valley in Chamoli District of Uttarakhand, India.

Urgam Valley (30°30′20.93′′ to 30°34′12.35′′N and 79°26′14.02′′ to 79°30′17.26′′ E) is located in north-eastern Chamoli district in Uttarakhand, India (Figure 1). The valley joins the Kalp Ganga Valley at 1,300 m amsl to the surrounding mountain tops above 3,000 m amsl. Crops consist mainly of three types, namely, Rabi, Kharif, and Zaid. The main Rabi crops of the region are Wheat and Mustard and Kharif crop are Rice, Maize, Finger millet, Barnyard grasses, and Amaranthus. Zaid crops include Beans, Cucumber, and pumpkin. Among the fruits are Apple, Peach, Cherry, and Walnut. Annual rainfall ranges from 2000 to 2500 mm while temperature ranges from 15 to 35°C during summer and -2 to 15°C in winter. Urgam Valley spans over a wide spread of topographic and climatic conditions, namely, alpine, subalpine, and temperate zones provide a range of plant habitats.

### 2.2. Field Survey and Data Collection

Local surveys including uses of medicinal plants of Urgam Valley were done between August 2015 and July 2016. Ninety-six informants were randomly selected in 11 villages. After giving prior informed consent, informants answered questionnaires (see the Appendix) in the local language (*Garhwali*), with photos of 110 medicinal plants as references. Answers were elicited based on plant species (“what do you know about [plants name]?” and based on disease condition (“which plants you use when suffering from [disease name]?”).

The questionnaires were then compiled detailed information for each plant on local name, life form, local uses, method of use or drug preparation, and amount of use (dose). Apart from the general population survey of villagers and shepherds, local male (Vaidyas) and female specialists (Daai) were also sought to compare their knowledge to that of the general population.

### 2.3. Plant Collection and Identification

Voucher specimens were prepared for the traditionally used plants documented in this study. Specimens were identified using Flora of District Garhwal [8] and Flora of Chamoli [9] and in comparison with the specimens of Garhwal University Herbarium, Srinagar Garhwal (GUH). Plant specimens were mounted on herbarium sheets and preserved in HAPPRC Herbarium. Plant names reported here were matched using The Plant List [10].

### 2.4. Data Analysis

#### 2.4.1. Comparing Consensus in Plant Use across Categories of Use

An informant consensus factor* (ICF) *was used to measure the consensus in plant use for a given illness treatment in the study area. To develop this consensus, all treated diseases were grouped into nine categories: (a) gastrointestinal disorders, (b) fever and aches, (c) diseases of the skin, (d) remove weakness, immunomodulator, anaemia, (e) ophthalmologic complaints, (f) poisonous bite, (g) dental problems, (h) ear ache, and (i) hearing problems. Within these categories, ICF was calculated according to the following formula [11]:

(1) $$\begin{array}{c} ICF=\frac{\left(Nur-Ntaxa\right)}{\left(Nur-1\right)}, \end{array}$$

where* Nur* refers to the number of use-reports for a particular ailment category and* Ntaxa* refers to the number of taxa used for a particular ailment category by all informants. ICF value ranges from 0 to 1. A high ICF value (close to 1.0) indicates “consensus” indicating relatively few taxa is reported by a large proportion of informants for an ailment category.

#### 2.4.2. Comparing Plants and Uses across Informants

To test whether the traditional medicinal plant knowledge varied with age, the total plants or total uses reported by each informant (excluding healers) were summed up and ran linear regressions and natural spline regressions, using the package splines [12] in the R statistical framework (Version 3.3.0).

To test whether healers reported a different set of plants and uses altogether, the 89 informants who had reported more than 10 species were compared. A matrix with plants as columns and informants were constructed as rows, calculated Bray-Curtis distances among each pair of informants based on how similar their answers were, and used nonmetric multidimensional scaling to plot informants based on these distances. To calculate the significance of specialist healer status, the fit of this factor on the location of informants in the ordination space was compared to that of 999 randomized shuffles using the R package vegan [13].

## 3. Results

### 3.1. Demographic Features of Informants

A total of 96 people were interviewed consisting of seven local healers from both female (Daai) and male (Vaidyas healers). Most (48 participants or 50%) were 41-60 years old with 27 informants or 28.1% were 40 years old and younger. Seventeen were illiterate, while 4 young practitioners held a tertiary education (degree/diploma) (Table 1).

### 3.2. Traditional Medicinal Plants Diversity

**Figure 2:**
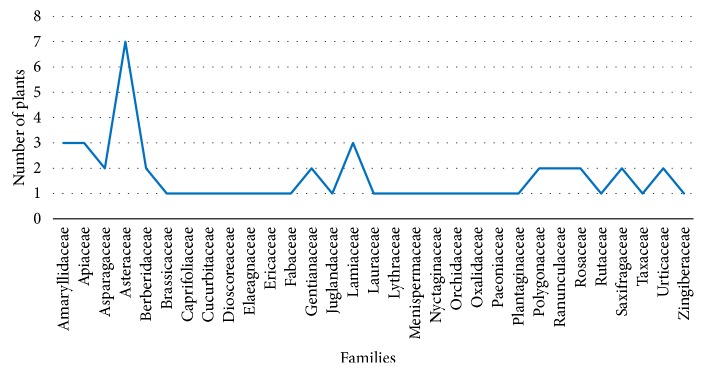
Number of medicinal plants in different families.

Fifty-one species representing 31 families are used by local inhabitants of Urgam Valley in Chamoli Garhwal for local health care (see Table 2). Out of 31 families, most (21 families) were dicotyledons, 9 were monocotyledons, and 1 was gymnosperm. The most represented families were asteraceae (7 species), followed by lamiaceae, amaryllidaceae, and apiaceae (3 species each) (Figure 2). Most species (39 species, 77%) were herbaceous plants, though trees (7 species, 14%), climbers (3 species, 6%), and shrubs (2 species, 4%) were also included.

### 3.3. Plant Part Use and Drug Preparation

**Figure 3:**
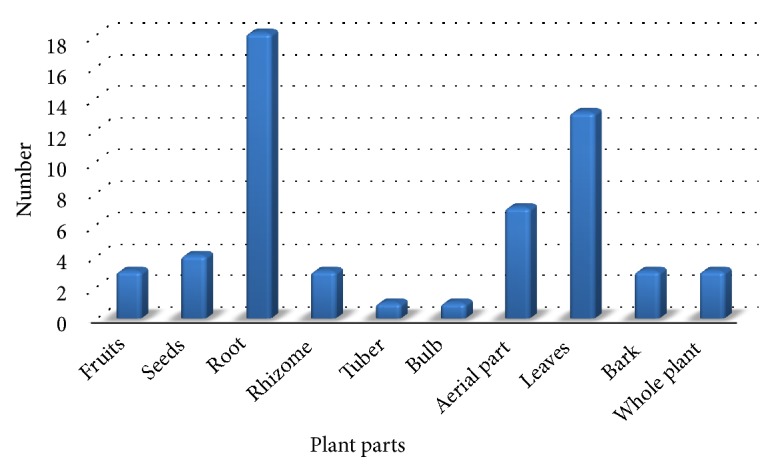
Plant parts used to cure different ailments by inhabitants of Urgam Valley.

Plant parts used were mostly roots (18 species, 32%) and leaves (13 species, 23%). Also recorded were aerial parts (7 species, 13%), seeds (4 species, 7%), fruits, rhizome, bark, and whole plants each two species (5%) (Figure 3).

**Figure 4:**
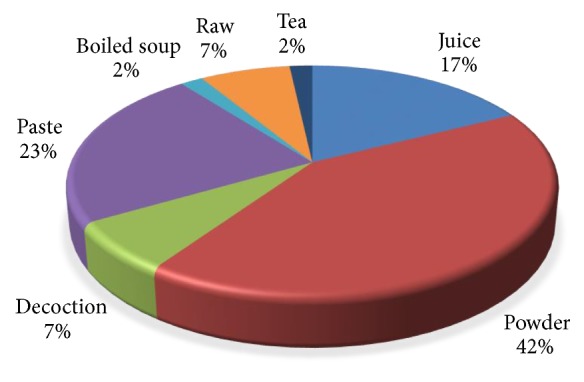
Traditional drug preparations by inhabitants of Urgam Valley.

The different type of formulations prepared by local inhabitants of Urgam recorded during the study was primarily plant powder (42% of formulations). Other preparations are paste (23%), extract/juice (17%), decoction and raw (7%), and herbal tea (2%) (Figure 4). All formulations are prepared by local practitioner (Vaidyas), elders, or those with more experience in herbal medicines.

### 3.4. Informant Consensus Factor

The highest consensus among informants (ICF) is found for* Aconitum balfourii *Stapf for poisonous bite (PB) followed by* Berberis aristata* DC. and* Berberis lycium *Royle for ophtalmologic complaints (OP), and* Potentilla lineata *Trevor for dental problems (DP) (0.99) (Table 3).

### 3.5. Comparing Plants and Uses across Informants

**Figure 6:**
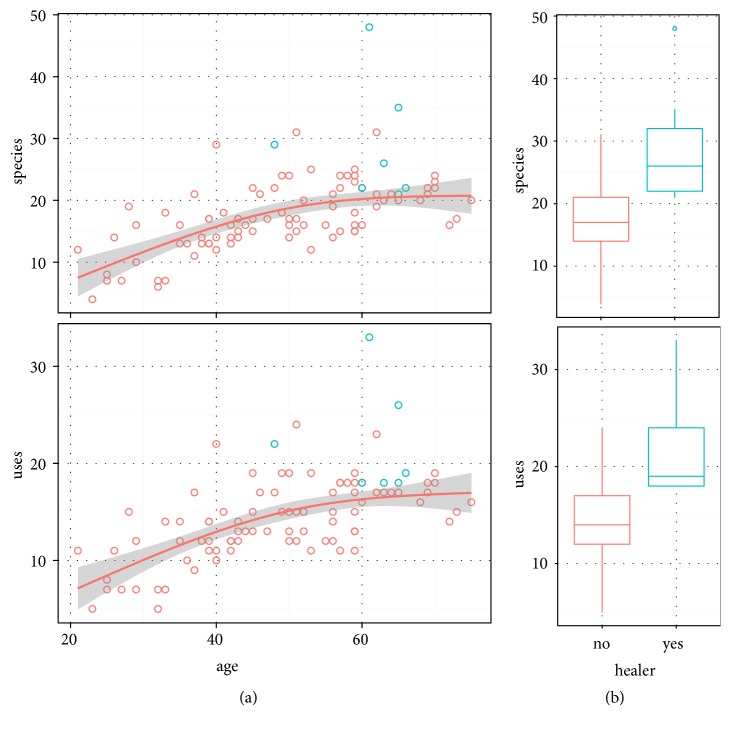
Species and uses reported by the general populace (red) increase with age (a) and are greatest for specialist healers (blue, b). The line indicates a natural spline regression in which the increase in knowledge with age flattens above age 50.

Inhabitants of Urgam Valley have a generally strong knowledge of medicinal plants, with informants reporting on average 18 plant species and 13 uses. This knowledge increases with age: linear regressions on age significantly increase for uses (y=0.18*∗*age+5.3, p<0.01, r-squared = 0.39) and for plant species (y=0.24*∗*age+5.5, p<0.01, r-squared = 0.37). The natural spline regressions show that this effect is less steep at higher ages (significantly nonlinear): that is, there is less increase in knowledge after about age 50 (Figure 6(a)). Healers, who were excluded from this analysis, report more plants and more uses than the average predicted value for their age (Figure 6(b)). Elders also tend more to report learning from their parents as a source of knowledge and tend to easily identify plants and their localities and characters, while some younger informants struggled to give information.

**Figure 7:**
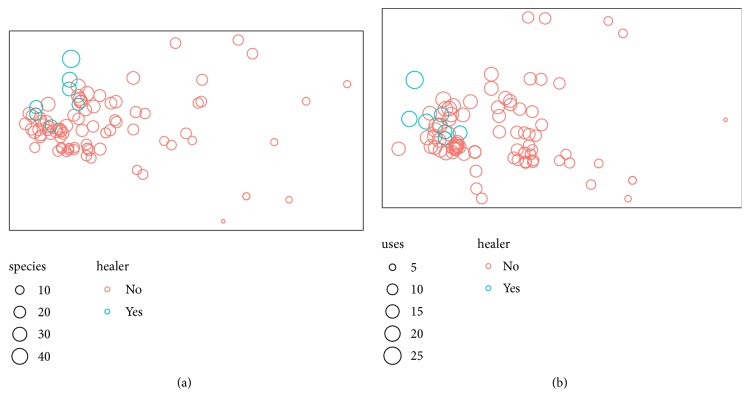
A nonmetric multidimensional scaling of points (informants) which are plotted closer together when species (a) or uses (b) reported are more similar and sized by the count for each informant of species or uses. Although there is overlap, healers (blue) occupy a significantly different section of the ordination spaces, showing that they report particular plants and uses.

Ordinations show similarity between informants by plotting those who reported more similar lists of plants or more similar lists of species are closer together (Figure 7). Although there is a great deal of overlap, specialist healers do report a significantly different set of plants (p=0.01, r-squared=0.06) and uses (p<0.01, r-squared=0.07) than nonspecialists. For instance,* Dioscorea bulbifera, Polygonatum verticillatum, Jurinea macrocephala,* and* Prunus persica *were only reported by healers;* Bergenia ciliata, Allium cepa, *and* Cinnamomum tamala *were more widely reported, but most frequently by healers (all healers reported these plants, compared to only <50% of nonhealers). Likewise, infection after breakage of hair in body “Baaltod” was only reported by healers, and Control blood pressure' and “Ear ache” were reported more widely, but much more frequently by healers (all healers reported these uses, compared to <50% of nonhealers).

## 4. Discussion

Medicinal plants are globally used in local health care by ethnic communities of the world and the knowledge of folk medicine is being documented throughout the world.

Our results show strong consensus on plant uses in Urgam Valley, with high informant consensus values across all categories. Further, we show that knowledge of traditional uses and of medicinal plants is higher in elders (*bujurg*), who learnt this knowledge from their parents or forefathers and associated plant medicine with positive attitudes, but also with regular practice of identifying and using plants to treat different ailments. We also showed that specialists tend to report different and unique species and were associated with some species that were widely reported, but most consistently reported by specialist. For instance,* Bergenia ciliata *(Haworth) Sternberg was reported here used for stones by every healer. This is a widely used plant, with similar use citations reported locally [8, 105] but also across the greater Himalayan region for a variety of uses [106]

Most commonly mentioned plants across the general population have also been reported previously for similar uses from the region. For instance,* Picrorhiza kurrooa *Benth., which was reported by nearly every informant, is used for fever similar to Bhat et al. [107], where it was reported for fever and stomach ache.* Zanthoxylum armatum *DC., reported by 95 informants for cleaning teeth and toothache, was reported for similar uses locally [108] and more distantly by Abbasi et al. [109].* Berberis lycium *Royle DC., reported by 92 informants for conjunctivitis, was also reported by Gaur [8] for ophthalmia and Bhat et al. [107] for eye irritation.* Aconitum heterophyllum *Wall.ex Royle root powder, reported by 69 informants for stomach ache and fever, was also reported elsewhere [107, 108] for the same uses. More distantly, the species is also reported for dysentery [106] in Northern Pakistan.* Juglans regia *L., reported by 56 for cleaning teeth and treatment of skin diseases, was also reported for similar uses from Northern Pakistan [106, 110] while, in Uttarakhand, Gaur [8] reported its use as fishery, dye, fungicide, and insecticide.* Dactylorhiza hatagirea *(Don.) Soo., reported by 39 for cut and wounds and stomach ache, was also reported for similar uses locally [107].* Aconitum balfourii *Stapt., which was uncommonly reported for snake bites in this study, was reported previously for similar uses: use in poisonous skin diseases [107], as antidote of snake and scorpion sting, and for rheumatism, arthritis and paralysis from Nanda Devi Biosphere reserve [111], and leprosy [108].

## 5. Conclusion

The study suggests that while there remains a rich knowledge of medicinal plants in Urgam Valley, most knowledge is held by elders (*bujurg*) and specialist healers (*vaidyas *and* daai*). Knowledge of medicinal plants is important and frequently used by local inhabitants to support their health care. Pharmacological activity on most of the plants is yet unknown so medicinal plants use in Urgam might be helpful in new drug discovery and pharmacological properties. Most of the highly useful plants of Himalaya are threatened with overexploitation and irregular harvesting and now limited to few pockets.* Ex situ* and* in situ* conservation should be implemented to conserve biodiversity and these valuable medicinal plants. Cultivation, rather than wild-harvest, of threatened valuable medicinal plants may support the traditional uses documented here, while also protecting wild populations.

## Figures and Tables

**Figure 5:**
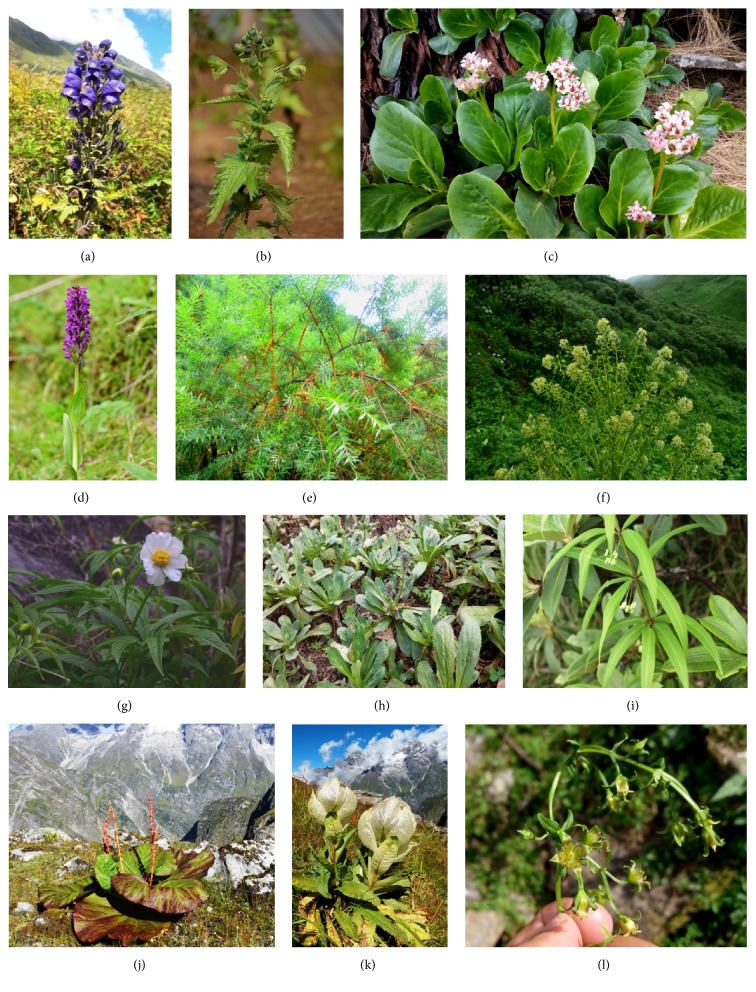
(a)* Aconitum balfourii;* (b)* Aconitum heterophyllum;* (c)* Bergenia stracheyi;* (d)* Dactylorhiza hatagirea;* (e)* Hippophae salicifolia;* (f)* Megacarpaea polyandra;* (g)* Paeonia emodi;* (h)* Picrorhiza kurrooa;* (i)* Polygonatum verticillatum;* (j)* Rheum moorcroftianum;* (k)* Saussurea obvallata;* (l)* Swertia chirayita*.

**Table 1:** Demographic characteristic of informants.

Variables	Categories	Number
Age	20-30	9

	31-40	17

	41-50	22

	51-60	26

	61-70	19

	71-80	3

Gender / specialist	Male (general)	72

	Female (general)	17

	Male specialist healer	4

	Female specialist healer	3

Education level	Illiterate	17

	1-5	27

	6-10	22

	10-12	26

	>12	4

Source of knowledge	By parents	71

	By other	20

	Self-experiments	5

Total		96

**Table 2:** Herbal medicine and mode of administration by inhabitants of Urgam Valley in Chamoli Garhwal, Uttarakhand, India.

S.N.	Scientific name, Family and collection number	Local name	Life forms	Parts used	Mode of preparation	Doses and administration	Diseases treated	UR	Pharmacological activity
1	*Aconitum balfourii* Stapf. Syn. *Aconitum lethale* Griff Ranunculaceae HAPPRC ASR 4103 Figure 5(a)	Meetha/Bhngwa	Herb	Root	Decoction (in cow urine), Paste	1/2 drop once a day	Snake bite	11	Diaphoretic, diuretic, analgesic, febrifuge, anti-inflammatory, anti-rheumatic, anti-pyretic, vermifuge, powerful sedative, narcotic and poison [14, 15]

2	*Aconitum heterophyllum* Wall.ex Royle Ranunculaceae HAPPRC ASR 4104 Figure 5(b)	Atees	Herb	Root	Juice, Powder	1/2 teaspoon with lukewarm water	Stomach ache, fever	69	Anti-inflammatory, antipyretic, Antibacterial, Immunomodulatory, Anthelminthic, Antihyperlipidemic, analgesic [14, 16–19]

3	*Ajuga parviflora* Benth. Lamiaceae HAPPRC ASR 4168	Neelkanthi	Herb	Whole plant	Powder or decoction	1/2-1 teaspoon 3 times a day with water	Stomach ache, fever	29	Hypertension, malaria, pneumonia, edema, as anthelmintic, antifungal, hypoglycemic, anti-inflammatory, antitussive, expectorant, antitumor and antimicrobial agents [20, 21]

4	*Allium cepa* L. Amaryllidaceae HAPPRC ASR 4120	Pyaj	Herb	Bulb	Juice	1-3 drops	Ear ache	48	Antitumor, antidiabetic, antioxidant, antibacterial, anti-allergic and molluscicidal activity [22, 23]

5	*Allium sativum* L. Amaryllidaceae HAPPRC ASR 4121	Lehsun	Herb	Whole plants	Paste	1/2 teaspoon	Burnt, Cut	23	Antibacterial, antiviral, antifungal, anti-parasitic, cardiovascular [24]

6	*Allium wallichii* Kunth. Amaryllidaceae HAPPRC ASR 4125	Lainka	Herb	Leaves	Powder	1/2 -1 teaspoon with water	Gastric	12	Anti-microbial, anti-oxidant, and anti-cancer [25]

7	*Angelica glauca* Edgew Apiaceae HAPPRC ASR 4146	Choru	Herb	Root	Powder	1/2 teaspoon with water	Gastric	9	Antioxidant, antimicrobial, and phytotoxic [26]

8	*Artemisia nilagirica* (C.B Clarke) Pamp. Asteraceae HAPPRC ASR 4136	Kunja	Herb	Leaves	Juice	1 teaspoon	Cut and wounds	24	Antimicrobial, antifungal, antibacterial, antifilarial, insecticidal, antiulcer, anticancer, antioxidant and anti-asthmatic [27]

9	*Asparagus filicinus *Buch.-Ham. ex. D.Don Asparagaceae HAPPRC ASR 4126	Jhirna	Herb	Root	Powder	1/2 -1 teaspoon with cowmilk	Remove weakness	13	Hypolipidemic [28]

10	*Berberis aristata* DC. Berberidaceae HAPPRC ASR 4163	Kingod	Shrub	Root	Decoction	1-2 drop	Eye ailments	87	Antimicrobial, antidepressant, diabetes mellitus, hepatoprotective, immunomodulatory [29]

11	*Berberis lycium *Royle Berberidaceae HAPPRC ASR 4164	Chotru	Herb	Bark	Decoction	1 teaspoon thrice a day	Diabetes, eye ailments	92	Antidiabetic, hepatoprotective, antihyperlipidemic, Antimicrobial, antimutagenic, pesticidal, wound healing [30]

12	*Bergenia ciliata* (Haw.) Sternb. Saxifragaceae HAPPRC ASR 4112	Syalphadi	Herb	Root	Decoction	1 teaspoon once a day with lukewarm water	Stone	45	Anti-tussive, antiulcer, anti-neoplastic, antioxidant, antibacterial, hypoglycaemic [31, 32]

13	*Bergenia stracheyi* (Hook.f.& Thomson) Engl Figure 5(c). Saxifragaceae HAPPRC ASR 4113	Pashanbhed	Herb	Root	Decoction	1 teaspoon once a day with lukewarm water	Stone	61	Anti-arthritic, antimicrobial, [32]

14	*Centella asiatica* (L.) Urb. Apiaceae HAPPRC ASR 4174	Brahmi	Herb	Leaves	Juice, Powder	1/2 -1 teaspoon with water	Coolant	26	Stimulatory-nervine tonic, rejuvenant, sedative, tranquilizer and intelligence promoting property, antiepileptic, leprosy, antinociceptive and anti-inflammatory [33–35]

15	*Cinnamomum tamala* (Buch.-Ham.) T.Nees & Eberm. Lauraceae HAPPRC ASR 4169	Tejpat	Tree	Leaves, bark	Powder	1/2 -1 teaspoon with water	Control blood pressure	49	Antidiabetic, antibacterial, anti-ulcer, antimicrobial [36]

16	*Cirsium wallichii* DC. Asteraceae HAPPRC ASR 4138	Kanjelu	Herb	Root	Powder, Juice	1/2-1 teaspoon thrice a day with water	Fever	2	Antimicrobial and Antioxidant [37]

17	*Cucumis sativus* L. Cucurbitaceae HAPPRC ASR 4153	Kakdi	Climber	Seeds	Powder	1/2 teaspoon with water	Diuretic	40	Antimicrobial, Antioxidant, Hypo cholesterolemic [38]

18	*Curcuma longa* L. Zingiberaceae HAPPRC ASR 4165	Haldu	Herb	Rhizome	Paste	1/2 teaspoon twice a day	Cut and wounds	86	Anti-HIV, antioxidant, anti-inflammatory, anti-tumor [39]

19	*Dactylorhiza hatagirea* (D.Don) Soo Orchidaceae HAPPRC ASR 4162 Figure 5(d)	Hathajadi	Herb	Root	Paste, Powder	1/2 teaspoon	Cut and wounds, fever, stomach ache	39	Antibacterial, aphrodisiac, antipyretic [14]

20	*Dioscorea bulbifera* L. Dioscoreaceae HAPPRC ASR 4139	Tairu	Climber	Tuber	Powder	1/2 teaspoon with water	Coolant	3	Antihyperlipidemic, antitumor, antioxidant, anorexiant, analgesic, anti-inflammatory, plasmid curing, anti-diabetic and antihyperglycemic[40]

21	*Eupatorium adenophorum* Sprengel Asteraceae HAPPRC ASR 4157	Basya	Herb	Leaves	Juice	1/2 - 1 teaspoon	Cut and wound	29	Analgesic, antifungal [41, 42]

22	*Girardinia diversifolia* (Link) Friis Urticaceae HAPPRC ASR 4118	Kandali	Herb	Root	Powder	1/2-1 teaspoon twice a day with water	Fever	1	Antibacterial, antifungal [43]

23	*Hippophae salicifolia* D.Don Elaeagnaceae HAPPRC ASR 4140 Figure 5(e)	Amesh	Tree	Fruits	Juice	5-10 teaspoon in 1 glass water	Coolant	6	Antibacterial, antifungal, anticancer, anti-inflammatory, immunomodulatory, radio-protective, adaptogenic, anti-atherosclerosis, and anti-sterility [44]

24	*Juglans regia* L. Juglandaceae HAPPRC ASR 4150	Akhrot	Tree	Fruit peel	Paste	1/2 teaspoon	Skin diseases	56	Antioxidant, antimicrobial, anti-atherogenic, anti-inflammatory and antimutagenic properties [45–47].

25	*Jurinea macrocephala* DC. Asteraceae HAPPRC ASR 4116	Bishkandara	Herb	Root	Powder	1/2 teaspoon thrice a day water	Fever	2	Antioxidant and Antibacterial [48]

26	*Macrotyloma uniflorum* (Lam.) Verdc. Fabaceae HAPPRC ASR 4114	Gahat	Herb	Seeds	Boiled soup	1 bowl thrice a day	Stone	96	Hepatoprotective, anti-obesity, anticalcifying, antidiabetic, antimicrobial [49–53]

27	*Megacarpaea polyandra* Benth ex Madden Brassicaceae HAPPRC ASR 4111 Figure 5(f)	Barmolu	Herb	Root	Powder	1/2 -1 teaspoon twice a day with lukewarm water	Gastric	4	Not reported

28	*Mentha piperita* L. Lamiaceae HAPPRC ASR 4148	Pudina	Herb	Leaves	Paste	1/2 -1 teaspoon water	Coolant	9	Antimicrobial [54]

29	*Mirabilis jalapa* L. Nyctaginaceae HAPPRC ASR 4117		Herb	Leaves	Paste	-	Cut and wounds	12	Antimicrobial [55]

30	*Nardostachys jatamansi* (D.Don) DC Caprifoliaceae HAPPRC ASR 4156	Maasi	Herb	Rhizome	Powder	1/2 teaspoon thrice a day with lukewarm water	Jaundice	1	Tonic, laxative, diuretic, spasmodic hepatoprotective, cardio protective [56–58]

31	*Ocimum tenuiflorum* L. Lamiaceae HAPPRC ASR 4115	Tulsi	Herb	Leaves	Powder or raw	3-5 leaves with water	Fever, cough and cold	75	Antimicrobial, radio-protective, ant diabetic, anti-carcinogenic [59, 60]

32	*Oxalis corniculata* L. Oxalidaceae HAPPRC ASR 4133	Almodu	Herb	Aerial part	Paste	1/2 teaspoon	Boils	26	Anti-inflammatory, refrigerant and antiscorbutic, hypoglycemic, antihypertensive, antipsychotic, stimulant, chronotropic & inotropic effect [61–63]

33	*Paeonia emodi* Royle Paeoniaceae HAPPRC ASR 4172 Figure 5(g)	Chandra	Herb	Leaves	Juice	1 teaspoon thrice a day with water	Fever	87	Backache, dropsy, epilepsy,tonic, emetic, cathartic, blood purifier and colic, purgative [64]

34	*Picrorhiza kurrooa* Royle ex Benth. Plantaginaceae HAPPRC ASR 4105 Figure 5(h)	Kadwi	Herb	Root	Powder	1/2 teaspoon thrice a day with water	Fever	96	Immunomodulatory, cardiotonic, antipyretic, anthelmintic, laxative and anti-asthmatic, hepatoprotective, anticholestatic, anti-ulcerogenic, anti-asthmatic and immune-regulatory functions [65, 66]

35	*Polygonatum verticillatum* (L.) All Asparagaceae HAPPRC ASR 4127 Figure 5(i)	Mahamaida/Salampanja	Herb	Rhizome	Powder	1/2-1 teaspoon thrice a day with water	Fever	3	Anti-inflammatory, antimalarial, antipyretic, insecticidal, antibacterial, antifungal, antidiarrheal [67–70]

36	*Potentilla lineata* Trevir. Syn. Potentilla fulgens L. Rosaceae HAPPRC ASR 4173	Bajrdanti	Herb	Root	Powder	1/2 teaspoon	Cleansing teeth	26	Anthelmintic [71]

37	*Prunus persica* (L.) Batsch Rosaceae HAPPRC ASR 4177	Aaru	Tree	Seeds pericarp	Paste	1/2 teaspoon	Infection after breakage of hair (Baaltod)	2	Anthelmintic, insecticidal, sedative, diuretic, demulcent, expectorant, vermicidal and are used in leucoderma and in piles [72]

38	*Punica granatum* L. Lythraceae HAPPRC ASR 4142	Anar	Tree	Fruits	Raw	1 fruit	Anemia	8	Antimicrobial, anti-inflammatory, anti-diabetic, anti cancer [73, 74]

39	*Rheum moorcroftianum* Royle Polygonaceae HAPPRC ASR 4160 Figure 5(j)	Dolu	Herb	Root	Powder, paste	1/2 teaspoon	Internal injury, cut and wounds	24	Purgative,antimicrobial, anti-inflammatory [14, 75]

40	*Rhododendron campanulatum* D. Don Ericaceae HAPPRC ASR 4178	Syamru	Tree	Leaves	Paste with oil	1/2 -1 teaspoon	Skin disease	6	Analgesic, anti-inflammatory [76]

41	*Rumex nepalensis* Spreng. Polygonaceae HAPPRC ASR 4167	Khuldya	Herb	Root	Powder, paste	1/2 -1 teaspoon thrice a day with lukewarm water	Pneumonia, Cut and wounds	1	Antioxidant, antitumour, anti-inflammatory, purgative,[77–79]

42	*Saussurea costus* (Falc.) Lipsch. Asteraceae HAPPRC ASR 4109	Kuth	Herb	Root and leaves	Paste	-	Cut and wounds	27	Anti-inflammatory, anticancer, hepatoprotective, antimicrobial [80, 81]

43	*Saussurea obvallata* (DC) Edgew Asteraceae HAPPRC ASR 4110 Figure 5(k)	Kaunl	Herb	Aerial part	Raw	-	To keep at home for increasing immunity	29	Antioxidant, antimicrobial [82]

44	*Selinum vaginatum* (Edgew.) C.B. Clarke Apiaceae HAPPRC ASR 4144	Bhutkesh	Herb	Root	Powder	1/2 teaspoon with water	Coolant	2	Antibacterial [83]

45	*Swertia chirayita *(Roxb.) Buch.-Ham. ex C.B.Clarke Gentianaceae HAPPRC ASR 4154 Figure 5(l)	Chiraitu	Herb	Whole plant	Powder	1/2-1 teaspoon thrice a day with water	Fever, stomach ache	78	Antibacterial, antifungal, antileishmania, antimalaria, anti-inflammatory, antidiabetic, hepatoprotective, antiviral [84–88]

46	*Swertia ciliata *(D.Don ex G.Don) B.L.Burtt Gentianaceae HAPPRC ASR 4166	Chirata	Herb	Aerial part	Powder	1/2 teaspoon with water	Fever, stomach ache	12	Antifungal [89]

47	*Tagetes erecta* L. Asteraceae HAPPRC ASR 4147	Gainda	Herb	Leaves	Juice	1-2 drops	Ear ache	1	Antipyretic, analgesic and anti-inflammatory [90]

48	*Taxus wallichiana* Zucc. Taxaceae HAPPRC ASR 4151	Thuner	Tree	Bark	Tea	1 cup once a day	High blood pressure	25	Immunomodulatory, anti-bacterial, anti-fungal, analgesic, anti-pyretic and anti-convulsance activities, anti-cancer [91, 92]

49	*Tinospora sinensis* (Lour.) Merr. Syn. Tinospora cordifolia (Willd.) Miers Menispermaceae HAPPRC ASR 4132	Giloe	Climber	Aerial part	Juice	1 teaspoon with water	Fever, Stomach ache	87	Anti-cancer, immunomodulatory, anti-diabetic, anti-toxicity [93–95]

50	*Urtica dioica* L. Urticaceae HAPPRC ASR 4130	Kandali	Herb	Aerial part	Raw/vegetable	-	Anaemia, remove weakness	81	Antidiabetic, hepatoprotective, antiviral, antimicrobial, anticancer, immunomodulatory [96–101]

51	*Zanthoxylum armatum* DC. Rutaceae HAPPRC ASR 4107	Timru	Shrub	Seeds, Stem or aerial part	Powder	1/2 teaspoon	Cleansing teeth and tooth ache	95	Anti-inflammatory, antibacterial, antifungal [102–104]

S.N.: serial number, Syn.: synonym, UR: use reports.

**Table 3:** Informants consensus factor for different ailment categories.

Ailment category	Number of use reports (Nur)	% of use reports	Number of taxa (Nt)	% of taxa	Informants consensus factor (ICF)
Gastrointestinal disorders	271	14.46	11	21.56	0.96

Fever and aches	580	30.94	13	25.49	0.97

Diseases of the skin	318	16.96	10	19.60	0.97

Remove weakness, immunomodulator, anaemia	131	6.99	4	7.84	0.97

Ophthalmologic complaints	179	9.55	2	3.92	0.99

Poisonous bite	11	0.58	1	1.96	1

Dental problems	121	6.45	2	3.92	0.99

Ear ache	49	2.61	2	3.92	0.97

Hearing problems	74	3.94	2	3.92	0.98

Others	140	7.47	4	7.84	0.97

Total	1874				

**Table 4:** 

S.No	Disease (Local name)	Disease (English name)
1	Anidra	Insomnia

2	Ankh ki bimariyan	Eye problems

3	Aankh aana	Eye flue

4	Apach	Indigestion

5	Baal jhadna	Hair fall

6	Baaltod	Boils after breakage of hair

7	Bukhar	Fever

8	Daant dard	Tooth ache

9	Diabetes	Diabetes

10	Gum chot	Wounds

11	Haddi tootna	Bon fracture

12	Jalna	Burnt

13	Jodo ka dard	Joint pain

14	Jukam	Cold

15	Kaan dard	Ear ache

16	Kamjori	Nutritive

17	Katna/Katyon	Cuts

18	Khasi	Cough

19	Makra/Daad	Herpes

20	Paichis	Dysentery

21	Pathri	Stone

22	Peelia	Jaundice

23	Pet dard	Stomach ache

24	Pet ke keede	Stomach worms

25	Phati Biwain	Feet crack

26	Phode, funsi	Boils

27	Pradar	Leukorrhea

28	Sar dard	Head ache

29	Syalbai	Kind of fever

30	T.B	Tuberculosis

**Table 5:** 

No.	Botanical name	+ =Yes - =No
1	*Aconitum balfourii* syn. *Aconitum lethale* Griff.	

2	*Aconitum heterophyllum* Wall. ex Royle	

3	*Aconitum violaceum* Jacquem. ex Stapf	

4	*Aconogonon rumicifolium* syn. *Pleuropteropyrum rumicifolium* (Royle ex Bab.) Munshi & Javeid	

5	*Acorus calamus* L.	

6	*Aesculus indica *(Wall. ex Cambess.) Hook.	

7	*Ajuga parviflora* Benth.	

8	*Allium cepa* L.	

9	*Allium sativum* L.	

10	*Allium stracheyi* Baker	

11	*Allium wallichii* Kunth	

12	*Angelica archangelica* L.	

13	*Angelica glauca* Edgew.	

14	*Arisaema tortuosum* (Wall.) Schott	

15	*Arnebia benthamii *(Wall. ex G.Don) I.M.Johnst.	

16	*Asparagus filicinus* Buch.-Ham. ex D.Don	

17	*Barleria cristata* L.	

18	*Berberis aristata* DC.	

19	*Berberis chitria *Buch.-Ham. ex Lindl.	

20	Bergenia ciliata (Haw.) Sternb.	

21	*Bergenia stracheyi *(Hook. f. & Thomson) Engl.	

22	*Betula utilis* D. Don	

23	*Boehmeria rugulosa* Wedd.	

24	*Cedrus deodara *(Roxb. ex D.Don) G.Don	

25	*Centella asiatica* (L.) Urb.	

26	*Cinnamomum tamala *(Buch.-Ham.) T.Nees & Eberm.	

27	*Cirsium wallichii* DC.	

28	*Citrus aurantiifolia* (Christm.) Swingle	

29	*Cucumis sativus* L.	

30	*Curcuma longa* L.	

31	*Cynodon dactylon* (L.) Pers.	

32	*Dactylorhiza hatagirea* (D.Don) Soó	

33	*Delphinium vestitum* Wall. ex Royle	

34	*Dioscorea bulbifera* L.	

35	*Drymaria cordata *(L.) Willd. ex Schult.	

36	*Duchesnea indica* (Jacks.) Focke	

37	*Echinochloa frumentacea* Link	

38	*Eleusine coracana* (L.) Gaertn.	

39	*Eupatorium adenophorum* Spreng Syn. *Ageratina adenophora* (Spreng.) R.M.King & H.Rob.	

40	*Ficus palmata* Forssk.	

41	*Fritillaria roylei* Syn. *Fritillaria cirrhosa* D.Don	

42	*Geranium wallichianum* D.Don ex Sweet.	

43	*Girardinia diversifolia* (Link) Friis	

44	*Habenaria intermedia* D.Don	

45	*Hedera nepalensis* K.Koch	

46	*Hedychium spicatum* Sm.	

47	*Hippophae salicifolia* D.Don	

48	*Juglans regia* L.	

49	*Jurinea macrocephala* DC.	

50	*Lyonia ovalifolia* (Wall.) Drude	

51	*Macrotyloma uniflorum* (Lam.) Verdc.	

52	*Malaxis muscifera* (Lindl.) Kuntze	

53	*Megacarpaea polyandra* Benth. ex Madden	

54	*Mentha *×* piperita* L.	

55	*Morina longifolia* Wall. ex DC.	

56	*Nardostachys jatamansi* (D.Don) DC.	

57	*Nicandra physalodes* (L.) Gaertn.	

58	*Ocimum tenuiflorum* L.	

59	*Oxalis corniculata* L.	

60	*Paeonia emodi* Royle	

61	*Paris polyphylla* Sm.	

62	*Persicaria capitata *(Buch.-Ham. ex D.Don) H.Gross	

63	*Picrorhiza kurrooa* Royle ex Benth	

64	*Podophyllum hexandrum* Syn. *Sinopodophyllum hexandrum* (Royle) T.S.Ying	

65	*Polygonatum verticillatum* (L.) All.	

66	*Potentilla fulgens* L. Syn, *Potentilla lineata* Trevir.	

67	*Pouzolzia hirta* Blume ex Hassk.	

68	*Primula denticulate* Sm.	

69	*Prunus cerasoides* Buch.-Ham. ex D.Don	

70	*Prunus persica* (L.) Batsch	

71	*Punica granatum* L.	

72	*Quercus leucotrichophora* A.Camus Syn. *Quercus oblongata* D.Don	

73	*Rheum austral* D. Don	

74	*Rheum moorcroftianum* Royle	

75	*Rhododendron campanulatum* D. Don	

76	*Roscoea alpina* Royle	

77	*Rubia cordifolia* L.	

78	*Rubus ellipticus* Sm.	

79	*Rumex hastatus* D.Don	

80	*Rumex nepalensis* Spreng.	

81	*Satyrium nepalense* D.Don	

82	*Saussurea costus* (Falc.) Lipsch.	

83	*Saussurea gossypiphora* D.Don	

84	*Saussurea obvallata* (DC.) Edgew.	

85	*Selinum vaginatum* C.B. Clarke	

86	*Skimmia laureola* Franch.	

89	*Solanum americanum* Mill.	

87	*Solanum khasianum* C.B. Clarke	

88	*Solanum nigrum* L. Syn.	

90	*Stellaria media* (L.) Vill.	

91	*Swertia chirayita *(Roxb.) Buch.-Ham. ex C.B.Clarke	

92	*Swertia ciliata *(D. Don ex G. Don) B.L. Burtt	

93	*Swertia cordata *(Wall. ex G. Don) C.B. Clarke	

94	*Tagetes erecta* L.	

95	*Tanacetum longifolium* Syn. *Athanasia linifolia* Burm.F.	

96	*Taraxacum officinale* Syn. *T. campylodes* G.E.Haglund	

97	*Taxus wallichiana* Zucc.	

98	*Terminalia bellirica* (Gaertn.) Roxb.	

99	*Terminalia chebula* Retz.	

100	*Thalictrum foliolosum* DC.	

101	*Tinospora sinensis* (Lour.) Merr.	

102	*Trichosanthes tricuspidata* Lour.	

103	*Trillium govanianum* Wall. ex D.Don	

104	*Urtica ardens* Link	

105	*Urtica dioica* L.	

106	*Valeriana wallichii* DC. Syn. *Valeriana jatamansi* Jones	

107	*Vanda cristata* Wall. ex Lindl.	

108	*Viola canescens* Wall.	

109	*Zanthoxylum armatum* DC.	

110	*Zingiber officinale* Roscoe	

## Data Availability

The data used to support the findings of this study are available from the corresponding author upon request.

## Appendix

### Questionnaires

*Informants' Details*

1. What is your name?
2. Gender: Male/Female
3. How old are you?
4. What is your Education?: Illiterate/5^th^/High school/Intermediates/graduation
5. What is your occupation?
6. Location/residence
7. Altitude
8. Do you know about medicinal plants? Yes/no
9. If yes
10. Which plants do you know?
    (8.1) Plant (Local name)
    (8.2) Habit (Tree/ Herb/ Shrub/Climber)
    (8.3) Number /name of disease (s) treated?
    (8.4) How you identify particular disease: Symptoms?
    (8.5) Plant part used (Root/leaves/ stem/ flowers/ fruit/ aerial part/whole plants)
    (8.6) Method of crude drug preparation and administration?
    (8.7) Dosage
    (8.8) How you collect and stored medicinal herbs and their preparation?
    (8.9) How much time we can use these stored preparation? (About expiry date )
    (8.10) Have you ever used this or just you knowledge from forefather or elsewhere?
    (8.11) Other importance?
    (8.12) Cultivated/ Wild
    (8.13) Wild availability:Common/scattered/ rare/ very rare
    (8.14) Natural location: High altitude/ middle altitude/ lower altitude/ every where
    (8.15) Natural pockets where you seen or collected? (Place name)
    (8.16) Availability of particular medicinal plants increases or decreases?
      If increases/decreases
    (8.17) What is your opinion for why increase or decrease?
    (8.18) Conservation required? Yes/No
      If yes
    (8.19) How can we conserve these important species?

*Remarks*

  Plants identified as ………………………………………… (Botanical name and family)
  Signature of Researcher

*Informants' Consent*

  I..................................................... (Informants name) declare that information given by me is true, complete and accurate and I am fully consent for it.
  Date........................................ (Signature/Thumb impression of Informant)
  - * Photographic base survey (N=110)*
  - To show one by one photograph to informants and ask have ever saw this plants?
  - If informants know about plants (Repeat from (8.1) to (8.19))
  - * List of medicinal plants with local name*
  - What do you know about…. plants? (Local name)
  - If informants know about plants (Repeat from (8.1) to (8.19))
  - * Disease base information*
  - What do you know about….(diseases name)?
  - If they know and use some medicinal plant for particular diseases
  The diseases list used to collect information is shown in Table 4. (Repeat (8.1) to (8.19))
  Plant list (see Table 5)
